# Potential decline in carbon carrying capacity under projected climate-wildfire interactions in the Sierra Nevada

**DOI:** 10.1038/s41598-017-02686-0

**Published:** 2017-05-25

**Authors:** Shuang Liang, Matthew D. Hurteau, Anthony LeRoy Westerling

**Affiliations:** 1 Intercollege Graduate Degree Program in Ecology and Department of Ecosystem Science and Management, The Pennsylvania State University, University Park, PA, USA; 2 Department of Biology, University of New Mexico, Albuquerque, NM USA; 3 Sierra Nevada Research Institute, University of California, Merced, CA USA

## Abstract

Ecosystem carbon carrying capacity (CCC) is determined by prevailing climate and natural disturbance regimes, conditions that are projected to change significantly. The interaction of changing climate and its effects on disturbance regimes is expected to affect forest regeneration and growth, which may diminish forest carbon (C) stocks and uptake. We modeled landscape C dynamics over 590 years along the latitudinal gradient of the U.S. Sierra Nevada Mountains under climate and area burned by large wildfires projected by late 21^st^ century. We assumed climate and wildfire stabilize at late-21^st^ century conditions (2090–2100) to facilitate analysis of lags between warming and changing CCC. We show that compared with historical (1980–2010) climate and wildfire conditions, projected scenarios would drive a significant decrease of up to 73% in mean total ecosystem carbon (TEC) by the end of the 590-year simulation. Tree regeneration failure due to intensified growing season dryness and increased area burned would substantially decrease forested area, transitioning the system from C sink to source. Our results demonstrate the potential for a lower CCC in the system due to extensive vegetation type conversion from forest to non-forest types, and suggest a decline in the contribution of Sierra Nevada forests to U.S. C sink.

## Introduction

Changes in climate and wildfire regimes may initiate cascading effects on forest successional trajectories and cause substantial changes in the CCC and C sink strength of the Sierra Nevada Mountains^1–5^. Climate-driven tree mortality, especially of large trees that contribute disproportionately to C uptake and storage, could lead to a weakening of forest C sequestration^6, 7^. Although a delayed response to changing climate by overstory trees and compensatory growth among species may mediate the immediate impacts on forest C^8^, large changes in the spatial distribution of dominant species’ regeneration driven by changing temperature and precipitation distributions is anticipated to cause extensive vegetation type conversion^9, 10^, which could profoundly affect ecosystem C uptake and system-level CCC in the long-term.

Increasing temperatures portend a future where prolonged fire season and reduced snowpack increase the frequency of large wildfires, accelerating the decline in C stock and sink of the system^11^. Biomass burning and decomposition of fire-killed trees can transition a forest from C sink to source for decades and the transition back to C sink depends on post-fire succession^12^. However, on-going climate change and increasing area burned may exert a significant impact on tree regeneration through reduced propagule availability and climatic constraints on seedling establishment^5, 13, 14^. If post-fire succession leads to a non-forested condition, we can expect reduced C sink and storage capacity^15, 16^.

**Figure 1:**
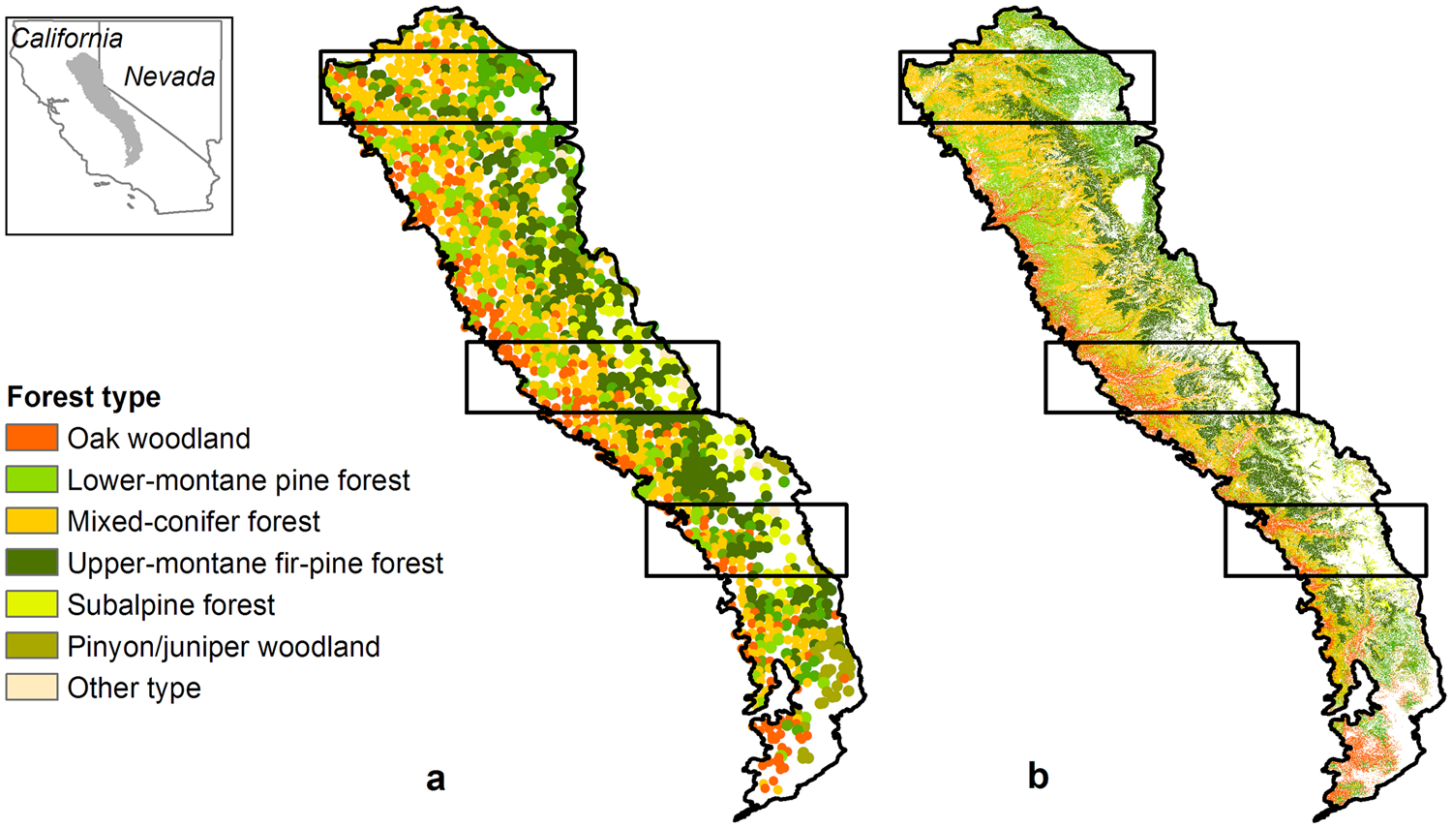
Spatial distribution of simulation transects. The polygons show the three transects simulated in the northern, central and southern Sierra Nevada, USA. (**a**) Distribution of forest types derived from FIA plots. (**b**) Initial distribution of simulated forest types derived from FIA data (see supplementary material). Maps were created using ArcGIS 10.1 (www.esri.com/software/arcgis).

To investigate the potential impacts of changing climate and wildfire regimes on long-term successional trajectory and forest C dynamics in the Sierra Nevada Mountains we simulated forest dynamics under projected future climate and area burned by large wildfires using LANDIS-II, a process-based, forest landscape model^17^. We selected three transects along the Sierra Nevada mountain range to capture the elevation and precipitation gradient (Fig. 1 and Supplementary Fig. S5). The median latitude of each transect was north: 39.875°, central: 37.876°, south: 36.749° and elevation range (north: 275–2591 m, central: 252–3978 m, south: 290–4388 m) of each transect increased from the north to the south. We ran simulations using historical (1980–2010) climate and wildfire conditions (baseline) and projected climate and wildfire conditions (projected) over a 590-year period to allow assessment of long-term successional change and C dynamics. The projected scenarios used climate projections from three general circulation models forced with a business-as-usual emission scenario and their associated area burned by large wildfires (>200 ha) projections for 2010–2100 and assumed climate and area burned stabilized at late-century (2090–2100) conditions for 500 years beyond 2100 to facilitate analysis of lagged effects between ecosystem response and environmental change. Our simulations only focused on tree species and did not include non-tree species (see Supplementary materials and Discussion). We evaluated changes in total ecosystem C (TEC, Mg C ha^−1^, a measure of CCC), net ecosystem C balance (NECB, g C m^−2^ yr^−1^, net C flux that accounts for loss from wildfire), and forested area to quantify the effects of projected climate and wildfire conditions on forest succession and C dynamics.

## Results and Discussion

**Figure 2:**
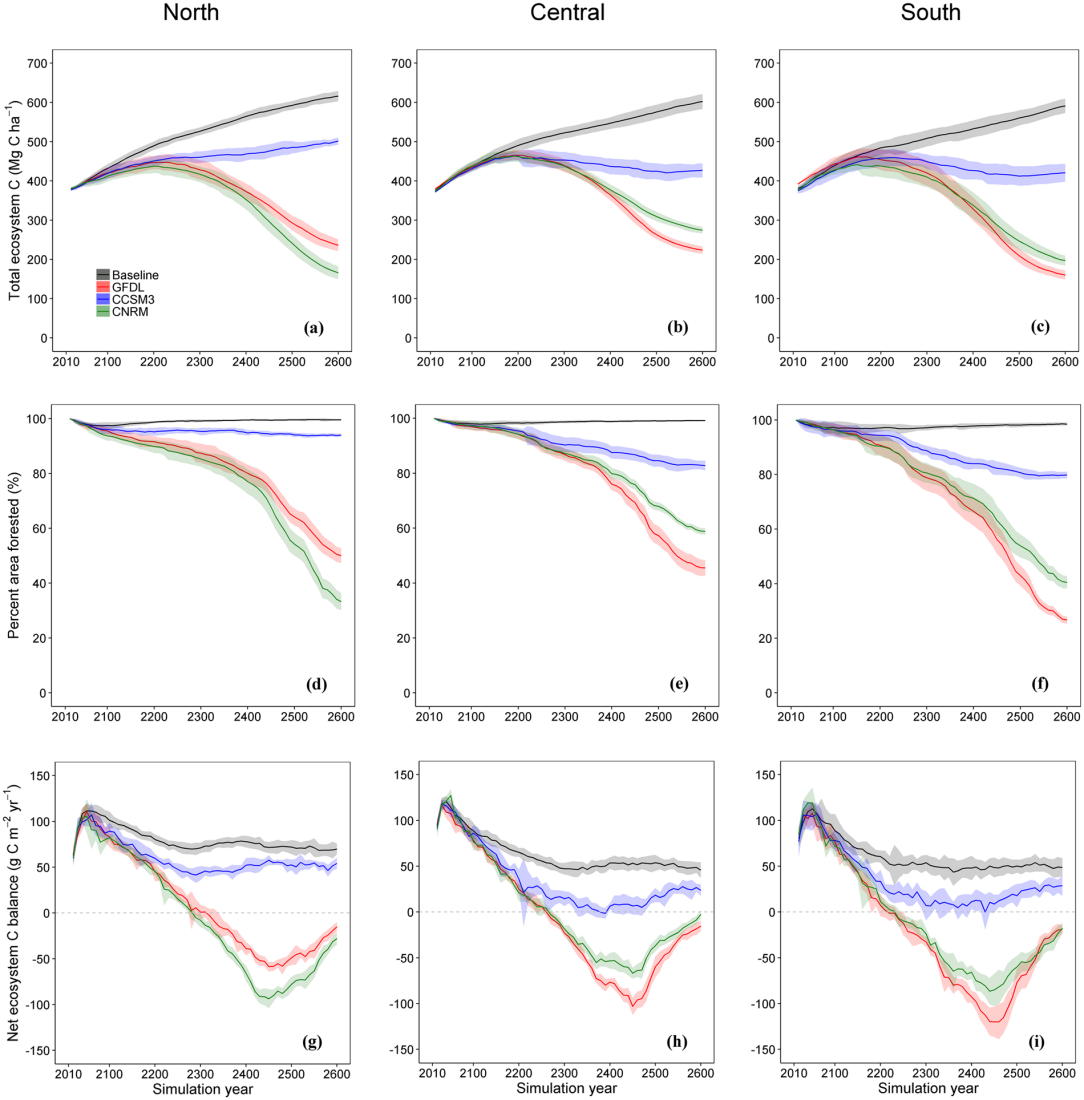
Changes in carbon stocks, fluxes, and forest cover. Mean total ecosystem C (**a–c**), percentage of area forested (**d–f**), and net ecosystem C balance (**g–i**) for baseline climate and wildfire and projected climate and wildfire scenarios under three general circulation models over the 590-year simulation period. The three column panels are for the three transects simulated in the northern, central, and southern Sierra Nevada. Shaded areas represent the standard deviations derived from replicate runs. See Supplementary Fig. 4 for spatial distribution of the results.

All three transects showed substantial decreases in mean TEC under projected climate and wildfire scenarios relative to the baseline, with the end-of-simulation decrease ranging from −19% to −73% across transects and GCMs (Fig. 2a–c). The trend in TEC became divergent among GCMs after ca. 300 years of simulation and the end-of-simulation TEC was substantially lower than the initial conditions under two GCMs that are relatively drier^10^. Greater area burned in the projected scenario (Supplementary Fig. 1) affected ecosystem C directly through mortality and indirectly by limiting tree regeneration due to the increasing distance to seed source, albeit nutrient release following fire may facilitate the growth of remaining trees. Potential post-fire tree regeneration was also climate limited as the niche space for regeneration and mature individuals of any given species were no longer coincident, a mismatch that can cause regeneration failure^9^. While area burned increased in all transects, with the largest increase occurring in the northern transect (Supplementary Fig. 1), the interactive effect of more area burned and changing climate caused a 72–82% decrease in mean total number of tree regeneration events over the simulation period relative to baseline (Supplementary Fig. 3). Decreased tree regeneration coupled with increasing extent of high-severity burns resulted in a 6–73% decrease in forested area across transects and GCMs by the end of simulation (Fig. 2d–f), suggesting a lack of forest development with succession and implicitly representing the potential vegetation type conversion from forest to non-forest type. The reduction in forest cover was most extensive at mid-elevation under drier GCMs (Supplementary Fig. 4), where current forest communities are predominantly comprised of drought-intolerant, fire-sensitive species and forest ecosystems are particularly sensitive to rising temperature and changes in snow cover^18^.

**Figure 3:**
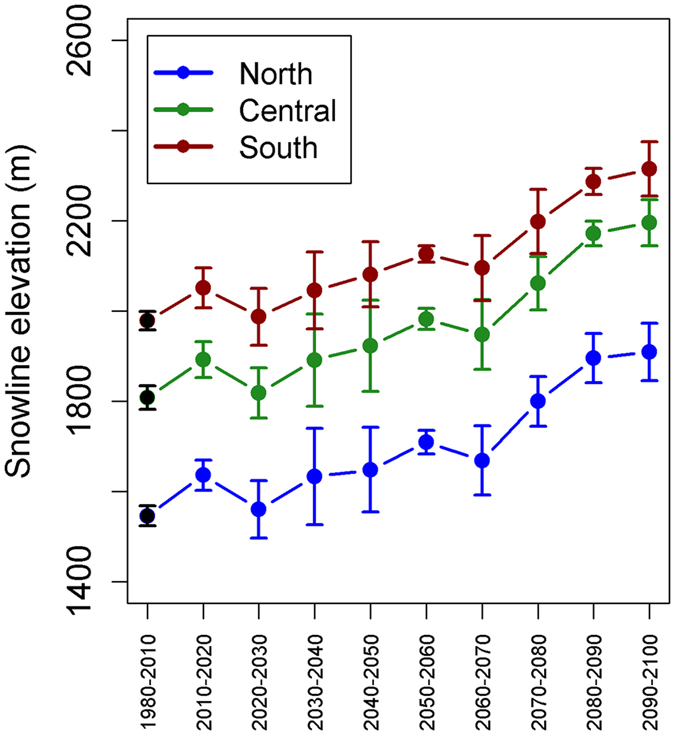
Overall change in snowline elevation. Values plotted are mean snowline elevations (threshold elevation below which monthly T ≥ 0 °C for whole year and consistent snow cover is absent) simulated under baseline climate (1980–2010, black dots) and projected climate (2010–2100, colored dots) for the three transects. Snowline elevations were determined by fitting linear regressions between mean monthly temperature of the coldest month and mean elevation of each climate grid cell (see Supplementary Fig. 6). The corresponding elevation where the regression line crossed the line that temperature equals zero was used as the snowline elevation. Error bars show the standard deviation derived from simulations across general circulation models and replicate runs.

**Figure 4:**
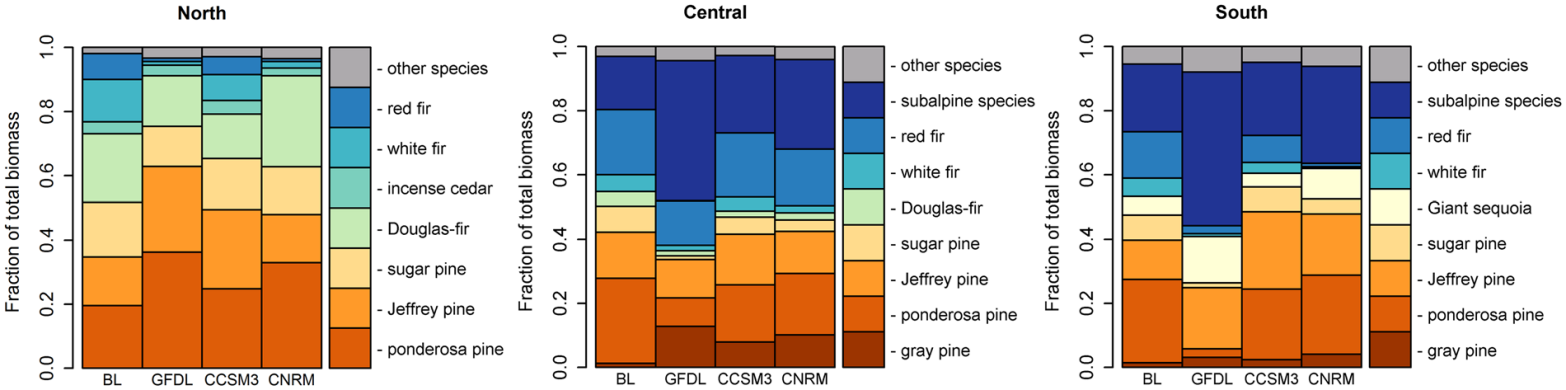
Distribution of aboveground carbon among dominant tree species. Mean fraction of aboveground carbon by dominant tree species at the end of the 590-year simulation under baseline climate and wildfire scenario (BL) and projected climate and wildfire scenario of each model (GFDL, CCSM3, CNRM) across the three transects. Values are means across replicate runs.

In this Mediterranean climate, where the majority of precipitation currently falls as snow during winter months, moisture released from melting snowpack is crucial for maintaining growth and regeneration in the growing season. Relative to baseline, increasing temperature under projected climate resulted in an increasing trend of upslope movement in snowline elevation (threshold elevation below which mean monthly temperature ≥0 °C for all year) over the century (Fig. 3), decreasing the area maintaining snow cover by 20–84% across transects under mean projected late-century (2090–2100) climate. The absence of snow cover primarily occurred at mid-elevation. While warmer winter temperatures accompanied by a 7–11% reduction in precipitation across transects decreased growing season moisture, substantial increases in summer temperature contributed to increased evaporative stress and further exacerbated growing season dryness (Supplementary Fig. 2). In unburned areas, projected increasing dryness reduced tree growth. In burned areas, it also limited the suite of species capable of reestablishing. We expected these factors to influence the CCC of the system, and their effects are demonstrated by our results. Furthermore, as CCC declined, the retained live tree C was mostly maintained at higher elevations where moisture is non-limiting and wildfire is less frequent and in pine trees (e.g. *Pinus sabiniana*, *P*. *ponderosa*, and *P*. *jeffreyi*) that are of higher drought tolerance (Fig. 4).

In the absence of changing climate, we expected NECB to remain positive across the landscape (Fig. 2g–i) because the fraction of the landscape impacted by wildfire in any given year is relatively small and growth in undisturbed areas and areas recovering from wildfire is adequate to maintain C uptake, a result that has previously been demonstrated^19^. However, with the combined effects of increasing area burned and changing climate we expected a decrease in C uptake due to slower rates of post-fire recovery. On average, across all transects and GCMs, we found 18-65% of the area became a C source by simulation end, which more than doubled the C-source area under the baseline scenario (9–23%). While each of the transects transitioned to a net C source for the latter half of the simulation period under the two drier GCMs (Fig. 2g–i), the timing of the sink-to-source transition occurred asynchronously, in a sequence from the southern to the northern Sierra. This result corresponded to decreasing precipitation with decreasing latitude (Supplementary Fig. 5) and the steeper decline in forested area in the southern Sierra (Fig. 2d–i). Furthermore, as the rate of decline in TEC began to stabilize after ca. 400 simulation years (Fig. 2a–c), NECB increased, suggesting that the C loss from wildfire and ecosystem respiration and C uptake from growth and regeneration were approaching an equilibrium state with the prevailing climate and natural disturbance conditions.

Despite the increasing temperature and moisture stress early in our simulations, our results, similar to other studies^19^, showed a trend of continued C uptake and increasing TEC into the 22^nd^ century (Fig. 2). This suggests that extensive harvesting over the past centuries has left a profound legacy effect on the landscape. While post-harvest regeneration and in-growth during a century of fire suppression have increased forest density, most stands are still young and well below their potential maximum C stock, facilitating continued C accumulation^19^. Furthermore, there often exists a time lag between ecosystem response and environmental change^8^ and ecosystems can be committed to change long before any appreciable difference emerges and can continue to change long after environmental change stabilizes^20^. On the other hand, the wildfire size distributions, used to determine maximum fire size, were based solely on climate and did not incorporate changing vegetation feedbacks into fire simulations^3^. It is possible that fire frequency and size may decrease in some areas as disturbance and changes in regeneration alter the biomass available to burn. However, most of the forest area we considered here maintains adequate fuel to carry large fires by the end of the 21^st^ century, and the development of a shrub layer following the decline in forest cover can sustain the spread of fire by increasing the fuel continuity over the landscape. While we did not simulate shrub community development, the fuel type associated with areas of no forest cover accounted for vegetation type conversion from forest to non-forest, allowing for fires to spread through non-forested areas. Despite this uncertainty, our results suggest forest ecosystems in the Sierra Nevada could require several hundred years to equilibrate to the new CCC, with the time required being a function of the rate and magnitude of change and the CCC level attained being a function of the variability in climate-wildfire interactions (Fig. 2).

The equilibration of forest C dynamics with changes in climate and wildfire regimes is unlikely to be unidirectional. The geographic disparity between climate space and species suitable to occupy that space can create a lagged effect for C dynamics. In our simulations, the acute effects of wildfire provided the catalyst for species change. However, extreme climate events could also serve as a catalyst for change. Hotter droughts have caused widespread conifer mortality in the southwestern US and are projected to occur with greater frequency as climate warms^7, 21^. These extreme events are already altering canopy density in the southern Sierra^22^ and could increase the rate and magnitude of change in forest C dynamics by further increasing the distance to potential seed sources and by removing the large individual trees that contribute disproportionately to the C sink. Furthermore, any process that reduces tree canopy cover in these ecosystems can increase shrub and herbaceous cover, which can further limit tree establishment through competition^13^. Increasing shrub and grasslands tend to burn severely in subsequent fires^16^ and may enhance local warming and drying, conditions that are detrimental to successive forest development.

Declining CCC in the Sierra Nevada under projected climate and wildfire has implications for both the global C cycle and policy goals for the State of California. Assuming TEC stabilizes at a level consistent with our lowest end-of-simulation values (Fig. 2a–c), mean ecosystem C loss from the entire Sierra Nevada would be as large as 663 Tg C (mean ∆TEC = −195 Mg C ha^−1^ across transects relative to current level) relative to current condition, which equals ca. 78% of the total aboveground C stock estimated for the state of California in 2010^23^. Over the latter half of the simulation period, the peak C flux to the atmosphere was 3.6 Tg C yr^−1^ (mean NECB = −105.4 g C m^−2^ yr^−1^ across transects), which is approximately 2% of the total conterminous US forest C sink in recent decades^24^ and is ca. 3% of California-wide GHG emissions in 1990 (116 Tg C), a 2020 emission reduction target for statewide emissions. Thus, our results suggest that the current climate buffering capacity of Sierran forests could transition to a liability if we maintain a business-as-usual global emissions trajectory.

It is important to note that our results are conservative because they do not account for the full suite of factors that can alter forest C dynamics. Our simulations only included climate-driven changes in wildfire size, which represents one attribute of a fire regime. Climate-driven increases in ignition frequency and the frequency of severe weather could accelerate changes in landscape C dynamics due to fire^4, 25^. In addition, our study did not include shrub species in the simulation due to the lack of inventory data and associated computational costs. However, given the potential for shrub communities to competitively dominate a site and create a positive feedback with subsequent fire that reinforces shrub dominance^16^, inclusion of tree-shrub interactions in the simulation may accelerate the decline in forest cover and C storage observed in this study. Furthermore, a continuation of recent drought-induced forest dieback could result in an acceleration of our simulated C dynamics over the coming decades, which would likely expedite the timeline for the transition to C source. Increasing temperatures and accompanying drought stress can also facilitate insect outbreaks by increasing survival and development rates of insect larva and by increasing the susceptibility of host species^26^. Western US wide, insect outbreaks accounted for a 6.1–9.3 Tg C yr^−1^ reduction in net ecosystem productivity through 2009 and insect-induced mortality can transition forests to a C source for decades in the most impacted areas^26, 27^.

Despite the large uncertainty associated with variability in projected climate and stochasticity in area burned, our results demonstrate the potential for large-scale vegetation type conversion from forest to non-forest types across the Sierra Nevada Mountains and suggest a steep decline in the capacity of the system to sequester C beyond the century. Given the projected late-century increase in fire probability for many forested areas in the northern hemisphere^4^, potential exists for the weakening of the forest C sink and decline in CCC over a substantial area where forest ecosystems are sensitive to changes in climate and wildfire.

## Methods

### Simulation model

To simulate future ecosystem C dynamics under projected climate and wildfire regimes we used the LANDIS-II forest succession and disturbance model. LANDIS-II is a process-based, spatially explicit landscape model built upon a core-extension modeling framework. The core interacts with extensions to integrate various ecosystem processes and disturbances that operate over time and space. We utilized three extensions to the LANDIS-II core: Century Succession extension (v3.1.1)^28^ which simulates carbon pools and fluxes, Dynamic Leaf Biomass Fuel extension (v2.0)^29^ which classifies generalized fuel types, and Dynamic Fire extension (v2.0.5)^29^ which implements stochastic wildfire events. In LANDIS-II, the landscape is divided into user-defined grid cells, which are classified into abiotically similar ecoregions. The gridded landscape is populated with initial communities of tree species represented by biomass in age-cohorts (see supplementary material). Successional processes of species cohort growth, competition and mortality are governed by species-specific life history and physiology that control dispersal, regeneration, and competitive ability.

The Century Succession extension simulates ecosystem C pools and fluxes as a function of species growth, climate, soil and their interactions based on the original CENTURY soil model^30^. However, the Century Succession extension does not account for rising atmospheric CO_2_ and the potential increase in water use efficiency^31^. While empirical evidence suggests an initial CO_2_ fertilization effect, other nutrients can become limiting^32^. The Dynamic Fire and Leaf Biomass Fuel extensions simulate stochastic wildfire as a function of parameterized fire regime attributes and forest-type delineated fuel types. The extensions account for changes in fuel characteristics that result from succession and disturbance (e.g. wildfire), and link fuel conditions with fire weather and topographic information to simulate fire behavior (e.g. rate of spread, direction). Simulated fire effects (proportion of cohorts killed) are dependent on both cohort age and species-specific fire tolerance such that the youngest cohorts with low fire tolerance are most vulnerable.

Model extensions were parameterized based on empirical data (e.g. forest inventory data, soil survey data, and historical wildfire records) and validated against other estimates in this region. Details can be found in the Supplementary Information. See Liang *et al*.^10^ for additional information and parameter values.

### Scenarios

Century Succession uses monthly averages and standard deviation of temperature and precipitation to create distributions for drawing monthly climate data used during simulations. For this study we used climate data from downscaled (12 km) climate projections based on three general circulation models (GCMs) and the *Special Report on Emissions Scenarios* (SRES) A2 emission scenario^33^ to calculate monthly means and standard deviations of climate variables for each decade from 2010 to 2100. The three GCMs, Geophysical Fluid Dynamics Laboratory coupled model (GFDL), National Center for Atmospheric Research Community Climate System Model (CCSM3), and Centre National de Recherches Météorologiques Coupled Global Climate Model (CNRM), better capture climate variability and seasonality over the historical period in California and could adequately span most of the spread in climate projections^33, 34^.

Dynamic Fire requires a user-defined fire size distribution to simulate area burned by wildfire. In this study, we built fire size distributions based on large wildfires (>200 ha). Although large wildfires that escape initial suppression effort represent a small fraction of total wildfires, they account for a disproportionately large fraction of annual area burned, and are largely influenced by climate at regional scales^11^. To develop fire size distribution parameters, we used climate projection-specific large wildfire (>200 ha) updated burned area projections (12 km, see supplementary material).

For each transect we simulated two types of scenarios over a period of 590 years that started from current conditions (2010) using a ten-year time step. We simulated the baseline climate and wildfire scenario, which assumes a continuation of the 1980–2010 climate and area burned, to quantify the effects of changing climate and area burned. To simulate projected climate and area burned, we developed monthly climate distributions and fire size distributions for each decade from 2010 to 2100 and used distributions from the last decade (2090–2100) for the years beyond 2100. Except for climate distribution and fire size distribution parameters, all other parameters were kept constant between scenarios. We ran ten replicates for each scenario to account for climatic variability and wildfire stochasticity.

## Electronic supplementary material

Supplemental Material

## References

[1] Keith H, Mackey BG, Lindenmayer DB (2009). Re-evaluation of forest biomass carbon stocks and lessons from the world’s most carbon-dense forests. Proc. Natl. Acad. Sci. USA.

[2] Seager R (2007). Model projections of an imminent transition to a more arid climate in southwestern North America. Science.

[3] Westerling AL (2011). Climate change and growth scenarios for California wildfire. Clim. Chang.

[4] Moritz MA (2012). Climate change and disruptions to global fire activity. Ecosphere.

[5] Stephens SL (2013). Managing forests and fire in changing climates. Science.

[6] Stephenson NL (2014). Rate of tree carbon accumulation increases continuously with tree size. Nature.

[7] McDowell NG (2016). Multi-scale predictions of massive conifer mortality due to chronic temperature rise. Nat. Clim. Chang..

[8] Svenning J, Sandel B (2013). Disequilibrium vegetation dynamics under future climate change. Am. J. Bot..

[9] Bell DM, Bradford JB, Lauenroth WK (2014). Early indicators of change: divergent climate envelopes between tree life stages imply range shifts in the western United States. Glob. Ecol. Biogeogr..

[10] Liang, S., Hurteau, M. D. & Westerling, A. L. Response of Sierra Nevada forests to projected climate-wildfire interactions. *Glob*. *Chang*. *Biol*. doi:10.1111/gcb.13544 (2016). 10.1111/gcb.13544 27801532

[11] Westerling AL (2016). Increasing western US forest wildfire activity: sensitivity to changes in the timing of spring. Phil. Trans. R. Soc. B.

[12] Dore S (2008). Long-term impact of a stand-replacing fire on ecosystem CO_2_ exchange of a Ponderosa pine forest. Glob. Chang. Biol..

[13] Collins BM, Roller GB (2013). Early forest dynamics in stand-replacing fire patches in the northern Sierra Nevada, California, USA. Landsc. Ecol..

[14] Harvey BJ, Donato DC, Turner MG (2016). High and dry: post-fire tree seedling establishment in subalpine forests decreases with post-fire drought and large stand-replacing burn patches. Glob. Ecol. Biogeogr..

[15] Hurteau MD, Brooks ML (2011). Short-and long-term effects of fire on carbon in US dry temperate forest systems. BioScience.

[16] Lauvaux CA, Skinner CN, Taylor AH (2016). High severity fire and mixed conifer forest-chaparral dynamics in the southern Cascade Range, USA. For. Ecol. Manage..

[17] Scheller RM (2007). Design, development, and application of LANDIS-II, a spatial landscape simulation model with flexible spatial and temporal resolution. Ecol. Model..

[18] Trujillo E, Molotch NP, Goulden ML, Kelly AE, Bales RC (2012). Elevation-dependent influence of snow accumulation on forest greening. Nat. Geosci..

[19] Loudermilk EL (2013). Carbon dynamics in the future forest: the importance of long-term successional legacy and climate–fire interactions. Glob. Chang. Biol..

[20] Jones C, Lowe J, Liddicoat S, Betts R (2009). Committed terrestrial ecosystem changes due to climate change. Nat. Geosci..

[21] Allen CD, Breshears DD, McDowell NG (2015). On underestimation of global vulnerability to tree mortality and forest die‐off from hotter drought in the Anthropocene. Ecosphere.

[22] Potter CS (2016). Landsat image analysis of tree mortality in the southern Sierra Nevada region of California during the 2013-2015 drought. J. Earth Sci. Clim. Change.

[23] Gonzalez P, Battles JJ, Collins BM, Robards T, Saah DS (2015). Aboveground live carbon stock changes of California wildland ecosystems, 2001–2010. For. Ecol. Manage..

[24] Pan Y (2011). A large and persistent carbon sink in the world’s forests. Science.

[25] Jolly, W. M. *et al*. Climate-induced variations in global wildfire danger from 1979 to 2013. *Nat*. *Commun*. **6**, 7537 (2015). 10.1038/ncomms8537 PMC4803474 26172867

[26] Ghimire B (2015). Large carbon release legacy from bark beetle outbreaks across Western United States. Glob. Chang. Biol..

[27] Kurz WA (2008). Mountain pine beetle and forest carbon feedback to climate change. Nature.

[28] Scheller RM, Hua D, Bolstad PV, Birdsey RA, Mladenoff DJ (2011). The effects of forest harvest intensity in combination with wind disturbance on carbon dynamics in Lake States Mesic Forests. Ecol. Model..

[29] Sturtevant BR, Scheller RM, Miranda BR, Shinneman D, Syphard A (2009). Simulating dynamic and mixed-severity fire regimes: A process-based fire extension for LANDIS-II. Ecol. Model..

[30] Parton WJ (1993). Observations and modeling of biomass and soil organic matter dynamics for the grassland biome worldwide. Glob. Biogeochem. Cycles.

[31] Keenan TF (2013). Increase in forest water-use efficiency as atmospheric carbon dioxide concentrations rise. Nature.

[32] Norby RJ, Warren JM, Iversen CM, Medlyn BE, McMurtrie RE (2010). CO2 enhancement of forest productivity constrained by limited nitrogen availability. Proc. Natl. Acad. Sci. USA.

[33] Cayan, D. *et al*. Climate change scenarios and sea level rise estimates for the California 2009 Climate Change Scenarios Assessment. California Energy Commission Report CEC-500-2009-014-D, California Climate Change Center, Sacramento, CA (2009).

[34] Pierce DW, Barnett TP, Santer BD, Gleckler PJ (2009). Selecting global climate models for regional climate change studies. Proc. Natl. Acad. Sci. USA.

