# Supplementary material for: Potential decline in carbon carrying capacity under projected climate-wildfire interactions in the Sierra Nevada
Source: Sci Rep. 2017 May 25;7:2420. doi: 10.1038/s41598-017-02686-0 (PMC5445098; doi:10.1038/s41598-017-02686-0)
Supplement: Supplementary file 1 — Supplemental Material [file 41598_2017_2686_MOESM1_ESM.pdf]

Supplementary information

Potential decline in carbon carrying capacity under projected climate-wildfire interactions in the Sierra Nevada

Shuang Liang, Matthew D. Hurteau, Anthony Leroy Westerling

Model extension and parameterization

We have previously calibrated and validated the model for the entire Sierra Nevada forests[1] and here we summarize our modeling approach.

The LANDIS-II core module requires an initial communities layer that represents the distribution of species age-cohorts across the landscape and an ecoregion layer that divides the landscape by similarity of edaphic and climatic conditions. We developed the initial communities layer by dividing the landscape into a 150m grid and assigning species age-cohorts to each grid cell. We used the spatially representative U.S. Forest Service Forest Inventory and Analysis (FIA) plot data[2] from 2000-2010 to derive tree species composition and age-cohort information within each plot. We summarized each plot into unique species-age cohorts using 10-year age bins and stratified the plots given a suite of attributes including forest type, ecoregion type, elevation range (+/- 100m), aspect, and county. We stratified the study area based on the same attributes that were obtained from spatial data layers[3-5] and assigned the FIA plots to grid cells based on the similarity of attributes. For each grid cell, the plot used to populate species-age cohorts was randomly selected from a set of candidate plots. The initial communities layer included 24 tree species (Table S1) and did not include non-tree species. To capture general patterns of vegetation, climate, and soil type and facilitate ecoregion-level parameter input, we divided the Sierra Nevada mountain range into 18 ecoregions using the U.S. Forest Service Ecological Provinces and Sections map[4] and the U.S. Environmental Protection Agency Level IV ecoregion map[6].

The Century extension requires parameters at the levels of tree species, functional groups, and ecoregions to model ecosystem C dynamics as a function of growth and establishment. Growth response, defined by growth sensitivity to temperature and water at the functional group level, is dependent on ecoregion-level soil characteristics and climate inputs. Species establishment ability, regulated by species life history traits and climatic envelopes for drought, growing degree days and minimum tolerable temperatures (Table S1), is influenced by climate. We leveraged species, functional groups, and ecoregion level parameters developed in previous study[1]. We used means and standard deviations of monthly temperature and precipitation from downscaled (12km) climate projections to create distributions for drawing monthly climate data for driving simulations. The scale of climate inputs was kept the same as the 12km climate projection grids to retain the spatial variability of climate over the landscape. The extension does not include the

fertilizing effect of CO_2 on tree growth. However sustained increase in water use efficiency with increasing atmospheric CO_2 is less likely due to nitrogen limitation[7, 8], especially given that nitrogen inputs in the Sierra Nevada are relatively small[9].

The Dynamic Fuel extension requires classification of forest communities into fuel types to represent general fuel conditions and influence wildfire behavior (e.g., rate of spread) and effects (e.g., fire severity). We employed 17 fuel types[1], which binned species that burn in a similar manner. Fuel type for a specific grid cell is reassigned each time step as a function of species composition and age and the occurrence of disturbance at the previous time step.

The Dynamic Fire extension, which is based on the Canadian Forest Fire Behavior Prediction System[10], requires parameterized fire regime attributes including fire size distribution and frequency, and representative fire weather and topographic data to simulate stochastic wildfire events based on fuel types. Each transect was stratified into three fire regions[1] using digital elevation model data to broadly reflect the elevation-delineated patterns of area burned and ignition that vary across low-elevation dry forests and woodlands (<1190m), mid-elevation mixed-conifer forests (1190-2120m), and upper montane and subalpine forests (>2120m). We built fire size distributions for each fire region using climate projection-specific area burned projections (12 km resolution) of large (>200 ha) wildfires. For the area burned projections we used the same modeling methodologies and data preparation that was employed in the Greater Yellowstone Area[11] to improve upon the California fire modeling[12], simulating wildfire burned area using three statistical models. Large fire presence/absence was modeled using a generalized linear model with a logit link with the glm() function in R (http://cran.r-project.org), estimating probabilities of fire presence as a function of climate, topography and fuels conditions (Fire Regime Condition Class, www.landfire.gov). Fire number conditional on fire presence was modeled with a Poisson Lognormal distribution fit to historical data and the same climate and biophysical site characteristics data. Fire size was simulated from a generalized Pareto distribution fit to historic fires and two covariates: cumulative monthly moisture deficit and fractional area with moderately to highly departed fire regime condition class. Using the same downscaled climate projections[11, 12] to simulate future wildfire activity, we used repeated draws from these distributions to characterize the distribution of burned area. We used these projection data for all 12 km grid cells within each fire region to develop fire size distributions. We calibrated the ignition frequency for each fire region based on contemporary wildfire records (http://frap.fire.ca.gov/). We used Remote Automatic Weather Stations data from stations that had the most complete fire season records for the period 2000-2013 to develop the representative fire weather distributions for each fire region. We obtained spatial layers of slope and aspect data (150 m resolution) from LANDFIRE (http://www.landfire.gov).

Model calibration and validation

We adjusted model parameters (e.g. functional group parameters defining growth sensitivity to temperature and moisture) to approximate seasonal growth patterns in representative forest types such as lower-elevation dry forests, mixed-conifer forests and subalpine forests with respect to growth seasonality in similar forest types at flux tower sites[13]. We compared populated initial communities and simulated aboveground biomass following model spin-up (where forest communities are grown to their parameterized ages, representing a current condition of the forested landscape) to FIA-derived data and other empirical-based estimates[14-16]. Comparisons can be found in Figure 1 and a previous study[1].

Supplementary References

1. Liang, S., Hurteau, M. D. & Westerling, A. L. Response of Sierra Nevada forests to projected climate-wildfire interactions. *Glob. Chang. Biol.* doi: 10.1111/gcb.13544 (2016).
2. O'Connell, B. M. *et al*. *The Forest Inventory and Analysis database: database description and users manual version 5.16 for Phase 2*. U.S. Department of Agriculture, Forest Service, Washington, DC (2013).
3. LANDFIRE Homepage of the LANDFIRE Project, U.S. Department of Agriculture, Forest Service, U.S. Department of Interior. Available online at http://www.landfire.gov. Accessed January 2013.
4. Cleland, D. T. *et al*. Ecological subregions: sections and subsections for the conterminous United States. U.S. Department of Agriculture, Forest Service, Washington, DC, Map scale, 1:3,500,000 (2007).
5. ESRI ArcMap desktop. Release 10.1. Environmental Systems Research Institute, Redlands, CA, USA (2012).

6. U.S. Environmental Protection Agency. Level III and IV ecoregions of the continental United States. National Health and Environmental Effects Research Laboratory, Corvallis, OR, Map scale 1:3,000,000 (2013).
7. Norby, R. J., Warren, J. M., Iversen, C. M., Medlyn, B. E. & McMurtrie, R. E. CO_2 enhancement of forest productivity constrained by limited nitrogen availability. *Proc. Natl Acad. Sci. USA* **107,** 19368–19373 (2010).
8. Keenan, T. F. *et al.* Increase in forest water-use efficiency as atmospheric carbon dioxide concentrations rise. *Nature* **499,** 324–7 (2013).
9. Fenn, M. E., Poth, M. A., Bytnerowicz, A., Sickman, J. O. & Takemoto, B. K. Effects of ozone, nitrogen deposition, and other stressors on montane ecosystems in the Sierra Nevada. *Dev. Environm. Sci.* **2,** 111–155 (2003).
10. van Wagner, C. E. *et al*. Development and Structure of the Canadian Forest Fire Behavior Prediction System. Fire Danger Group, Forestry Canada, Ottawa, Ontario (1992).
11. Westerling, A. L., Turner, M. G., Smithwick, E. H., Romme, W. H. & Ryan, M. G. Continued warming could transform Greater Yellowstone fire regimes by mid-21st Century. *Proc. Natl Acad. Sci. USA* **108,** 13165-13170 (2011a).
12. Westerling, A. L. *et al*. Climate change and growth scenarios for California wildfire. *Clim. Chang*. **109,** 445-463 (2011b).
13. Goulden, M. L. *et al*. Evapotranspiration along an elevation gradient in California's Sierra Nevada. *J. Geophys. Res.: Biogeosci.* **117,** G3 (2012).
14. Kellndorfer, J. *et al*. NACP aboveground biomass and carbon baseline data (NBCD 2000), USA, 2000. Data set. ORNL DAAC, Oak Ridge, Tennessee. Available online at daac. ornl. Gov (2011).

15. Wilson, B. T., Woodall, C. W. & Griffith, D. M. Imputing forest carbon stock estimates from inventory plots to a nationally continuous coverage. *Carbon Balance Manag.* **8,** 1-15 (2013).

16. Gonzalez, P., Battles, J. J., Collins, B. M., Robards, T. & Saah, D. S. Aboveground live carbon stock changes of California wildland ecosystems, 2001–2010. *For. Ecol. Manage.* **348,** 68–77 (2015).

Figure S1: Mean cumulative burned area over the course of simulation period in the northern, central and southern Sierra Nevada. Shading represents the standard deviation derived from simulations across general circulation models and replicate runs.

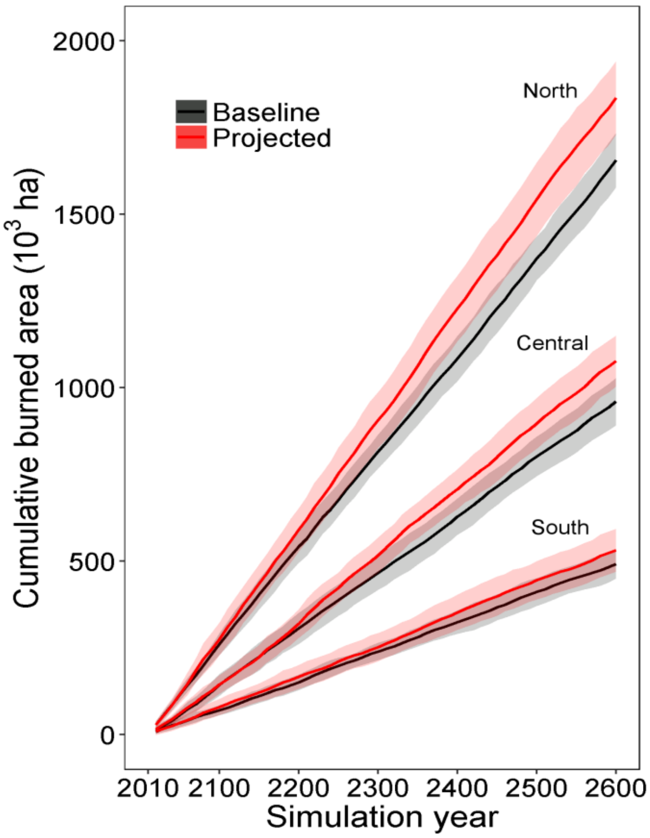

Figure S2: Mean temperature and precipitation for summer months (June-August) and winter months (December-February) under baseline climate and projected late-century (2090-2100) climate for each transect. Error bars show the standard deviation derived from model simulations.

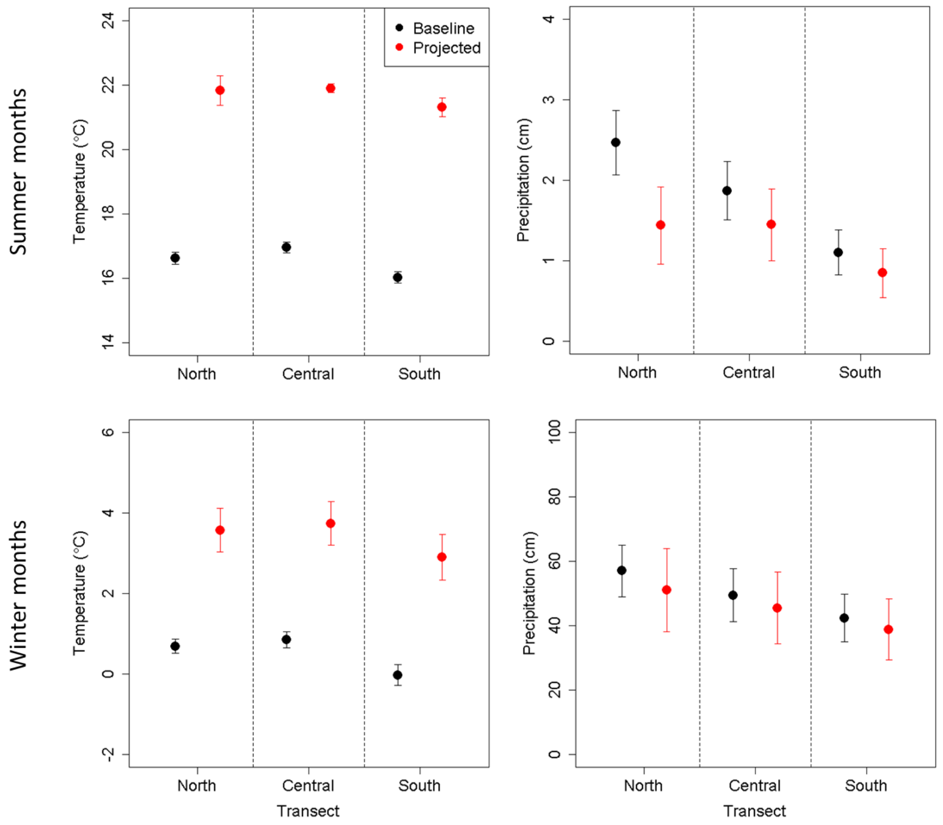

Figure S3: Percentage of total recruitment events over the simulation period under projected climate and wildfire scenarios relative to baseline at each transect. Error bars show the standard deviation derived from simulations across general circulation models and replicate runs.

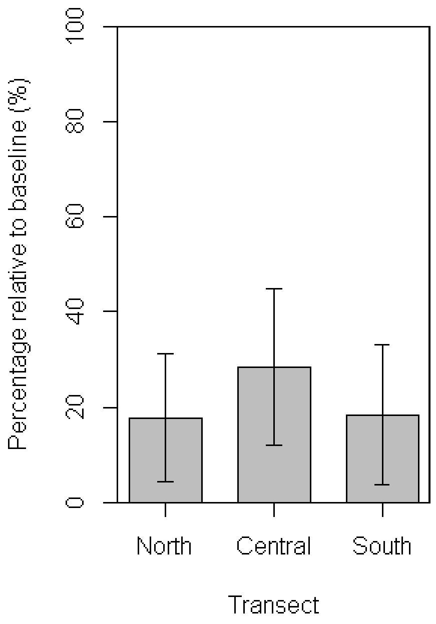

Figure S4: Spatial distribution of changes in forested area, total ecosystem C, and net ecosystem C balance from baseline to projected scenarios under each general circulation model by the end of the 590-year simulation in the three transects across the Sierra Nevada. Values are aggregated from the scale of forest simulations (150m) to the scale of the climate projections (12km) to account for the uniform climate data within each 12km grid cell and facilitate landscape-scale comparison between scenarios. The black lines stratify the landscape into low (<1190m), mid (1190-2120m) and high (>2120m) elevation bands from the northwest to the southeast. See Figure S5 for the spatial distribution of annual precipitation and elevation in the three transects as a reference. Maps were created using ArcGIS 10.1 (www.esri.com/software/arcgis).

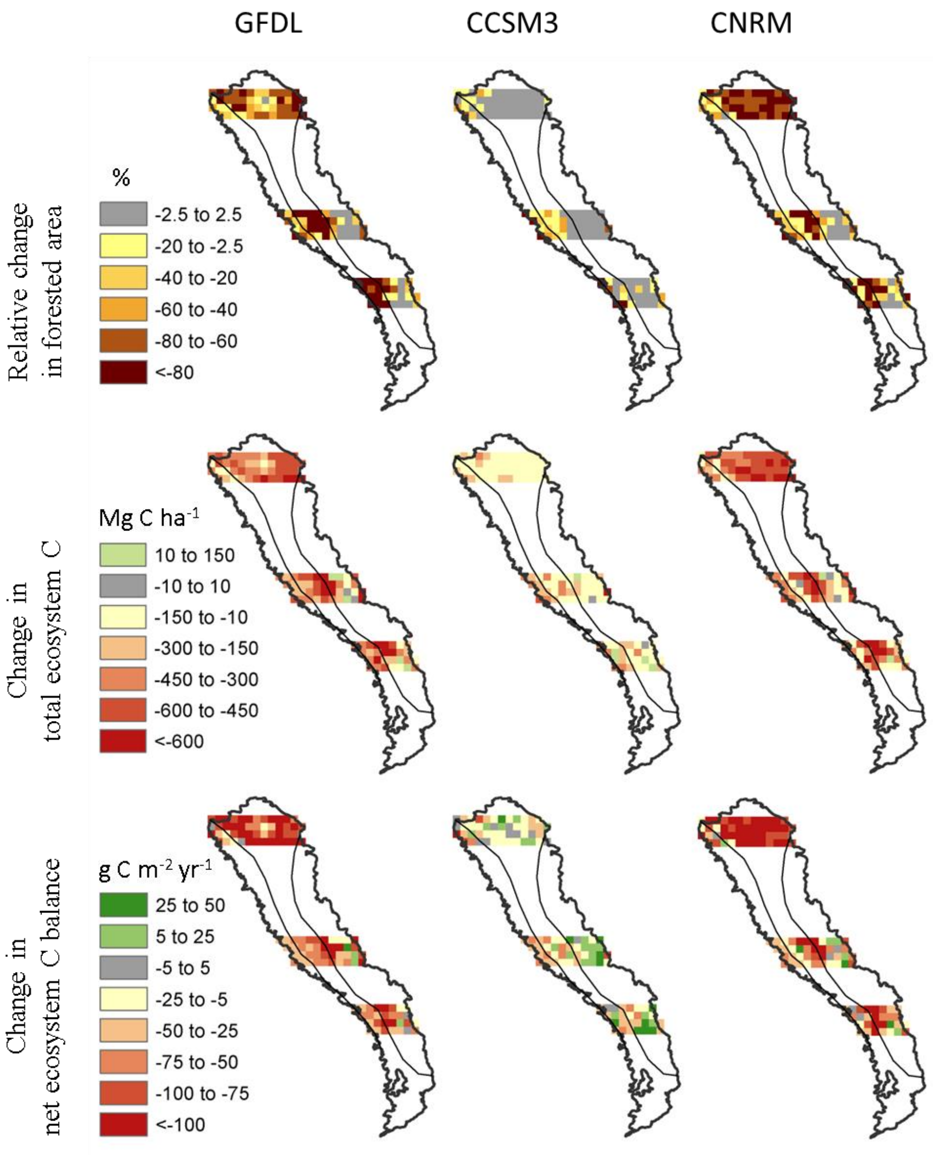

Figure S5: Spatial distribution of mean annual precipitation (1980-2010) and mean elevation in the three transects along the latitudinal gradient of the Sierra Nevada. Values are shown at the scale of climate projections (12km) and are averaged over the nested 150m grid cells within each 12km grid cell. Maps were created using ArcGIS 10.1 (www.esri.com/software/arcgis).

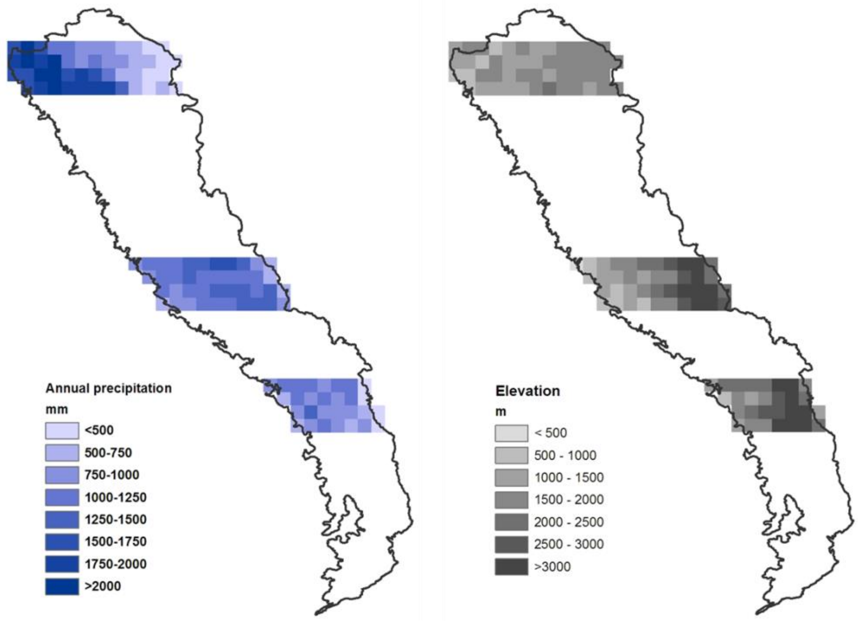

Figure S6: Example demonstrating the approach for determining snowline elevation. Each dot represents a climate grid within that transect. Red dots represent monthly temperature under projected climate and black dots are for baseline climate. Mean elevation for each climate grid is derived from Figure S5. Solid lines are linear regression lines. Corresponding elevation where regression line crosses the line that temperature equals zero is used as the snowline elevation.

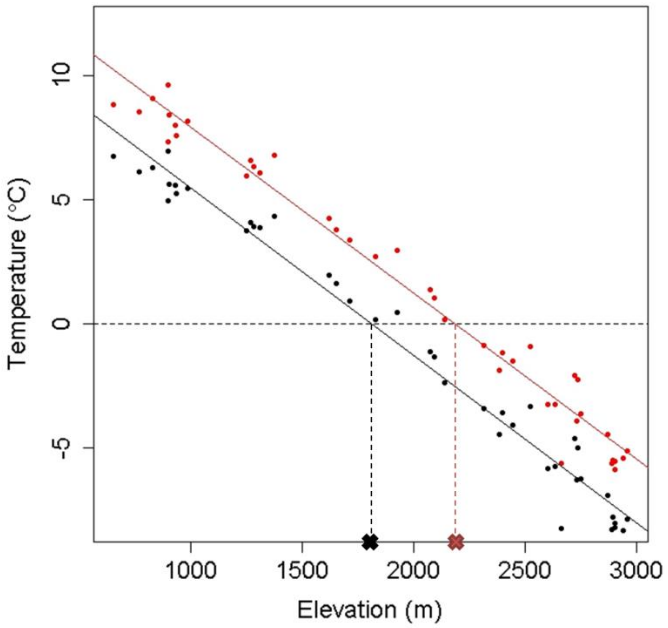

Table S1: Tree species and key life history traits and functional group attributes used in LANDIS-II modeling

Common name	Species name	Longevity (years)	Shade tolerance (1-5)	Fire tolerance (1-5)	Growing Degree Days Min	Growing Degree Days Max	Min. Jan. Temp. (ºC)	Max. Drought
White fir	*Abies concolor*	450	4	3	247	4031	-10	0.417
Red fir	*A. magnifica*	500	3	3	247	3582	-13	0.407
Sierra Juniper	*Juniperus occidentalis*	1000	2	1	247	3749	-14	0.42
Incense cedar	*Calocedrus decurrens*	550	4	3	332	4031	-8	0.424
Whitebark pine	*Pinus albicaulis*	900	2	2	247	2929	-17	0.385
Foxtail pine	*P. balfouriana*	1000	1	4	247	2937	-16	0.408
Jeffrey pine	*P. jeffreyi*	500	2	5	247	3993	-12	0.428
Sugar pine	*P. lambertiana*	550	3	4	285	4031	-8	0.422
Lodgepole pine	*P. contorta*	300	1	2	247	3582	-14	0.406
Limber pine	*P. flexilis*	1000	2	1	332	3176	-17	0.397
Western white pine	*P. monticola*	600	2	3	247	3582	-15	0.402
Ponderosa pine	*P. ponderosa*	400	2	5	232	4031	-8	0.43
Gray pine	*P. sabiniana*	200	1	3	1053	4641	-5	0.471
Singleleaf pinyon	*P. monophylla*	600	1	1	332	4008	-10	0.512
Douglas-fir	*Pseudotsuga menziesii*	750	3	3	332	4031	-6	0.413
Giant sequoia	*Sequoiadendron giganteum*	2000	1	5	364	3026	-9	0.458
Mountain hemlock	*Tsuga mertensiana*	800	5	1	247	2929	-15	0.386
Bigleaf maple	*Acer macrophyllum*	200	4	2	655	4031	-7	0.415
Pacific madrone	*Arbutus menziesii*	300	3	1	767	4031	-3	0.42
Quaking aspen	*Populus tremuloides*	175	1	2	258	3230	-16	0.421
Canyon live oak	*Quercus chrysolepis*	250	3	1	332	4031	-5	0.447
Blue oak	*Q. douglasii*	300	3	2	1157	4025	-5	0.49
California black oak	*Q. kelloggii*	300	3	2	332	4031	-6	0.433
Interior live oak	*Q. wislizeni*	200	3	1	608	4031	-5	0.437

Shade tolerance ranges from 1 to 5, with 1 being least shade-tolerant and 5 being most shade-tolerant.
Fire tolerance ranges from 1 to 5, with 1 being least fire-tolerant and 5 being most fire-tolerant.
Maximum drought index is defined in LANDIS-II as the fraction of drought days during the growing season that individual species can tolerate. A drought day is one in which soil moisture is below a critical soil moisture threshold (i.e., soil wilting point).
